# Review of Transducer Principles for Label-Free Biomolecular Interaction Analysis

**DOI:** 10.3390/bios1030070

**Published:** 2011-07-01

**Authors:** Martin Nirschl, Florian Reuter, Janos Vörös

**Affiliations:** 1 Laboratory of Biosensors and Bioelectronics, Institute for Biomedical Engineering, ETH Zurich, Switzerland; E-Mail: janos.voros@biomed.ee.ethz.ch; 2 Siemens Technology Accelerator GmbH, Otto-Hahn-Ring 6, 81739 Munich, Germany; E-Mail: florian.reuter@siemens.com

**Keywords:** biomolecular interaction analysis, BIA, sensor, transducer, drug discovery, drug development

## Abstract

Label-free biomolecular interaction analysis is an important technique to study the chemical binding between e.g., protein and protein or protein and small molecule in real-time. The parameters obtained with this technique, such as the affinity, are important for drug development. While the surface plasmon resonance (SPR) instruments are most widely used, new types of sensors are emerging. These developments are generally driven by the need for higher throughput, lower sample consumption or by the need of complimentary information to the SPR data. This review aims to give an overview about a wide range of sensor transducers, the working principles and the peculiarities of each technology, e.g., concerning the set-up, sensitivity, sensor size or required sample volume. Starting from optical technologies like the SPR and waveguide based sensors, acoustic sensors like the quartz crystal microbalance (QCM) and the film bulk acoustic resonator (FBAR), calorimetric and electrochemical sensors are covered. Technologies long established in the market are presented together with those newly commercially available and with technologies in the early development stage. Finally, the commercially available instruments are summarized together with their sensitivity and the number of sensors usable in parallel and an outlook for potential future developments is given.

## 1. Introduction

Biomolecular interaction analysis (BIA) is an important method for drug discovery and drug development [1]. Label-free sensors have the advantage that the adsorbed molecules do not require a chemical treatment like radioactive, fluorescent or other types of markers. The use of markers can be unproblematic when only the presence or quantity of a substance should be detected like in pregnancy tests. However, for the study of the interaction between molecules the presence of a label might alter the interaction process. In this case, the use of a label-free is a significant advantage and also saves resources. 

Several parameters are important when selecting a transducer to use for BIA: The most obvious is the limit of detection (LOD), which can be measured as the smallest detectable concentration of a certain substance or the lowest detectable molecular mass of a certain concentration of molecules, the lowest detectable affinity of a chemical reaction or for surface-based sensors the lowest detectable surface mass density. As only the transducer principles should be compared here, the smallest detectable surface mass will be focused on because this measure is only dependent on the transducer. Other parameters like the smallest detectable concentration depend highly on factors independent from the transducer like the used surface chemistry or the fluidic system. But other parameters besides the sensitivity are also equally important: The required sample volume is crucial if many substances or many different concentrations are measured like in high-throughput screening (HTS) or the sample volume is available in very limited amounts (e.g., human drug targets) or the transducer integrated into other processes delivering small sample amounts like on-bead screening [2]. The number of measurements that can be done at the same time with one sensor is important if a high throughput in a short time is desired. The more multiplexed a sensor is, the more parallel measurements can be performed without significantly increasing the equipment size and cost. A wide range of transducer principles were developed and used for BIA in the last decades. This section aims to give an overview of the state-of-the-art of different transducers used for label-free BIA. The most important parameters are summarized in Table 1. 

There are detailed reviews available for the acoustic [3,4], optical [5,6,7], electrochemical [8] and nanostructure-based [9,10] transducers. While there are also reviews about the most commonly used equipment and techniques used in BIA [11,12], this paper aims to give a more complete overview of both commercially available label-free transducers and transducers currently under development with a special emphasis on the transducer principles. 

Covering topics related to label-free biosensors, the reviews with a special emphasis on highly multiplexed technologies [13,14], microdispensing of liquids for biosensor arrays [15] and label-free cell-based assays in drug discovery [16] might be of interest to the reader. 

**Table 1:** Overview of the commercially transducer systems for Biomolecular interaction analysis (BIA). Information is taken from the website of the companies where not stated differently.

	Company name	Product name ^2^	Technology	Limit of detection [ng/cm^2^]	Number of parallel sensors	Sample volume ^6^	Sample volume per sensor/pixel ^7^	Web address	Comments
**SPR ^1^**	GE Healthcare	Biacore 4000	optical	0.01	16	60 µL (For 4 flow cells)	4 µL	www.biacore.com	
		Biacore T100		0.01	4	20 to 50 µL	21 to 50 µL		
**SPRi ^3^**	Horiba	SPRi-Plex™	optical	0.5	up to 1,000	1.6 mL	2 µL (target)/1.6 mL (ligand)	www.horiba.com	Up to 1,000 substances can be spotted, only one substance can be measured in flow
**BLI**	ForteBio	Octet RED384	optical	0.1	16	n/a	200 µL	www.fortebio.com	
**Diffraction Grating Based** ^**4**^	SRU Biosystems	BIND	optical	0.01	96-, 384- and 1,536-well microplate	n/a	down to 5 µL	www.srubiosystems.com	
	Corning	Epic		0.5	384-well microplate	n/a	15–30 µL typical	www.corning.com	
**Optical Waveguide Based**	MicroVacuum Ltd.	OWLS 210	optical	0.5	1	n/a	20 to 250 µL	www.owls-sensors.com	
	Farfield	*Ana*Light 4D		0.01	1	n/a	50 µL	www.farfield-group.com	
**ELM**	Maven Biotechnologies	LFIRE	optical	0.1	1	n/a	n/a	www.mavenbiotech.com	
**QCM ^5^**	Q-Sense	E4 Auto	acoustic	0.5	4	n/a	400 µL	www.q-sense.com	
**SAW**	SAW instruments GmbH	sam5	acoustic	0.05	5	40 to 80 µL	8 to 16 µL	www.saw-instruments.de	
**Electrochemical**	Eco Chemie	n/a		n/a	n/a	n/a	n/a	www.ecochemie.nl	
**ITC**	MicroCal	iTC_200_	calorimetric	n/a	1	n/a	n/a (at least 10 µg protein)	www.microcal.com	in-solution, no immobilization needed

^1^ Other SPR systems: Bio-Rad ProteOn XPR36 (www.bio-rad.com), Eco Chemie Autolab TWINGLE (www.ecochemie.nl), Reichert Inc. SR7000DC (www.reichertspr.com), Sierra Sensors GmbH (www.sierrasensors.com). ^2^ List of product is not complete, only the most sensitive products are listed. ^3^ Other SPRi systems: Biacore Flexchip (discontinued), Plerxera Bioscience PlexArray™ (www.plexera.com), GWC Technologies SPRimagerÆII (www.gwctechnologies.com), IBIS Technologies IBIS-iSPR (www.ibis-spr.nl). ^4^ Other diffraction grating based systems: Axela dotLab (www.axelabiosensors.com). ^5^ Other QCM systems: Sierra Sensors QCMA-1 (www.sierrasensors.com), TTP LabTech RAP (www.ttplabtech.com), Attana A200 (www.attana.com). ^6^ Sample volume means the minimum of sample volume required to follow one binding interaction. ^7^ The sample volume per pixel can vary from the overall sample volume if more than one pixel is in one flow cell.

## 2. Transducer Principles

### 2.1. Acoustic Sensors

Acoustic sensors comprise one or more vibrating elements that create acoustic waves. These waves can propagate on the surface, *i.e*., surface acoustic wave (SAW) or in the bulk of the resonator, *i.e*., bulk acoustic wave (BAW). These waves change their properties (e.g., amplitude or frequency) when molecules adsorb and physically or chemically bind to the sensor surface. This change is detected and contains information e.g., about the amount of adsorbed molecules. 

This overview of acoustic sensors is limited to acoustic sensors vibrating parallel to the sensor surface, as resonators vibrating vertically to the sensor surface (e.g., in the longitudinal mode) have a high loss of energy into the liquid and are limited in sensitivity and thus difficult to be used to monitor adsorbents of biomolecules in real-time. An overview over all acoustic microsensors including cantilever-based sensors or micromachined ultrasonic transducers (CMUTs) can be found in [17]. 

#### 2.1.1. Quartz Crystal Microbalance (QCM) and Quartz Crystal Microbalance with Dissipation Monitoring (QCM-D)

**Figure 1:**
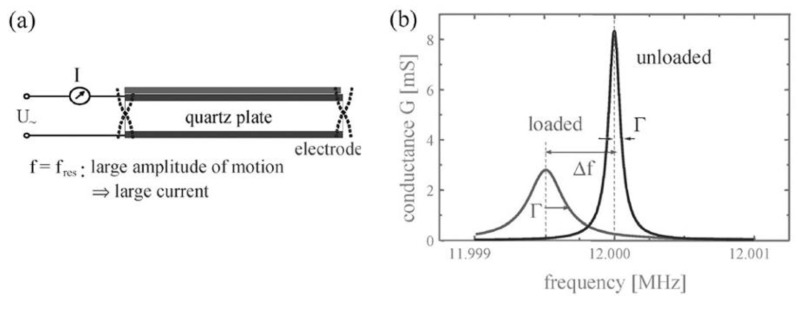
(**a**) Schematic diagram of the quartz crystal microbalance (QCM) and (**b**) the electrical characteristic with (loaded) and without (unloaded) adsorbed mass. From [22]—Reproduced by permission of the PCCP Owner Societies.

The QCM is a bulk acoustic wave (BAW) device, which consists of a piezoelectric quartz crystal, which resonates if it is electrically excited using two electrodes (Figure 1). Sauerbrey found that the resonance frequency decreases linearly if additional mass is attached to the sensor [18]. However, this is only true if the attached mass is rigid and small compared to the sensor mass. If the attached mass is not rigid, the viscoelastic properties have to be taken into account. This is mostly the case for operation in liquids [19] and for the adsorption of soft materials. With a model where the adsorbed soft material is represented by a viscous and an elastic element connected in parallel (*i.e.*, a Kelvin-Voigt material) under a Newtonian liquid it is possible to describe the frequency response also in liquid environment [20,21,22,23,24]. The frequency shift, which is influenced by the amount of attached mass, the liquid environment around the sensor and the viscoelastic properties of the adsorbent is hereby given by:

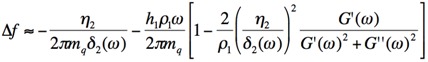
(1)
with 
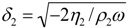
 where η is the viscosity, m the mass, ρ the density, *ω* the angular frequency, h the thickness of the adsorbent, G’ the storage and G’’ the loss modulus of the adsorbent. The index ‘1’ corresponds to the adsorbed layer, the index ‘q’ to the quartz and the index ‘2’ to the bulk liquid [25]. This model assumes that the viscosity of the adsorbent is constant over frequency, which is most likely not the case for most materials and should be therefore be carefully used especially if the measurement covers a broad range of frequencies [26]. 

The QCM has a LOD lower than 1 ng/cm^2^ and can also be used for adsorbents with several hundreds of nanometers thickness. Due to this high dynamic range the QCM is used in a broad application field, from small molecules up to cells [27]. 

More recently, attention was not only drawn to measuring the adsorbed mass but also to investigate the viscoelastic properties of the adsorbent. This can be done by not only reading out the resonance frequency, but also the motional resistance [28], the conductance [29] or the energy dissipation [30]. The latter system is named quartz crystal microbalance with dissipation monitoring (QCM-D). With this technique, novel types of investigations like on the changes of viscoelastic properties of polymers [22], vesicle adsorption and lipid bilayer formation [31], cross-linking of protein layers [32] and folding or unfolding of proteins were performed. 

In most commercially available QCM systems a sample volume of more than 50 µL is needed per flow cell, which motivates the search for a smaller BAW device with smaller sensor area. 

#### 2.1.2. Surface Acoustic Wave (SAW) Devices

**Figure 2:**
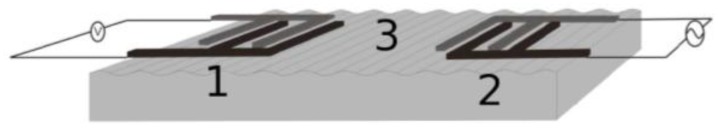
Typical set-up of a surface acoustic wave (SAW) biosensor: An acoustic wave propagates from a sender (**1**) to a receiver (**2**) passing the active sensor area (**3**) where its amplitude and velocity is influenced by the sensor surrounding (*i.e.*, liquid or adsorbed mass). Adopted from [40].

A SAW biosensor, schematically shown in Figure 2, consists of one or more interdigital transducers (ITD) built on a piezoelectric substrate, such as quartz (α-SiO_2_), lithium niobate (LiNbO_3_), or lithium tantalite (LiTaO_3_) [33]. The IDTs are interleaved electrodes that work as a sender to transfer electrical waves to acoustic waves and a receiver to transfer acoustic waves into an electrical signal. Between sender and receiver, the acoustic waves travel along the substrate, where the amplitude and velocity of the wave is influenced by adsorbed mass, viscoelastic changes and the conductivity of the surrounding liquid. 

In addition to this rather simple set-up, the IDTs might be covered with a protective layer to avoid corrosion of the metal electrodes in buffer solution or the sensitive area can be covered with a layer with low acoustic velocity (e.g., a polymer [34] or SiO_2_ [35]) in order to trap the wave close to the surface and minimize the energy dispersed into the substrate or the liquid. The effect of trapping the energy in a layer with an acoustic velocity lower than the surrounding is called the Love wave effect. Another way to confine the acoustic energy near the surface is to use a mass grating with a pitch of half the wavelength of the acoustic waves; the resulting waves are called shear horizontal waves (SHW). While there were many different device types tested for usage in biosensors, so called surface transverse waves (STW) or Love waves, or a combination of both seem to be most promising for a high performance sensor. An overview over the recent developments towards SAW biosensors can be found in [3]. 

The SAW devices can be structured using photolithography which allows to integrate a high number of sensors on a small area. Devices with fluidic volumes well below 1 µL have been developed [36]. SAW sensors have the highest theoretical mass sensitivity among the acoustic resonators [37] and with a shown limit of detection of lower than 0.08 ng/cm^2^ [38], a robust sensor system based on SAW would be extremely competitive to existing commercially available technology. The drawback of the SAW sensors is that it is difficult to build a robust device, because the frequency change is influenced by many factors like the conductance of the liquid and the conductance, dielectric and elastic constants of the adsorbent [39]. These perturbations make quantitative measurement challenging. 

#### 2.1.3. Film Bulk Acoustic Resonator (FBAR)

**Figure 3:**
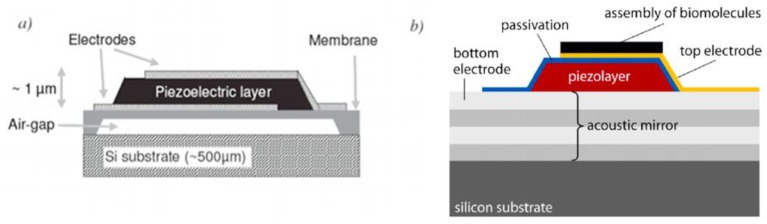
Film bulk acoustic resonators (FBARs) consist of a piezoelectric layer sandwiched between two electrodes over which the piezo layer is acoustically excited. The FBAR is isolated from the substrate by (**a**) an air gap or (**b**) an acoustic mirror. Reprinted from [55] and [56] with permission from Elsevier.

FBARs (Figure 3) are bulk acoustic wave (BAW) devices and operate in the thickness shear mode (TSM) like the QCM. However, while the QCM has been used for decades for the analysis of intermolecular interactions, FBARs have been produced just recently for the application in liquid [41,42,43,44]. Thin film bulk acoustic resonators vibrating in longitudinal mode have been produced before e.g., for filter applications [45]. For application in liquid, however, acoustic resonators operating in shear mode were developed, as the acoustic losses caused by longitudinal waves propagating into the liquid are too high to achieve sufficient Q-factors. Piezoelectric thin-films with the c-axis being inclined from the film normal were developed to achieve sufficiently high piezoelectric shear coupling coefficients [46,47,48,49,50,51,52,53]. While the working principle of FBAR and QCM is similar, the QCM is produced in a top-down and FBAR in a bottom-up process using thin-film technology. As a result FBARs can be made thinner, which results in a higher resonance frequency. FBARs operating from some hundreds of MHz to several GHz have been presented [45]. However, also determining the resonance frequency becomes more difficult for smaller devices, so that the noise increases. It was shown that the small size makes it possible to integrate many resonators on a small area [54]. This makes the FBAR especially promising for biomolecular interaction analysis with high throughput. 

### 2.2. Optical Sensors

The label-free optical biosensors introduced in this section are based on the interaction of light with the adsorbed biomolecules. Light is reflected at the active sensors surface where it is affected by the amount of adsorbent present at the surface. The adsorption of biomolecules can be quantified in real-time by monitoring the changes (e.g., intensity, wavelength, polarization, and phase) of the light after being reflected at the active sensor surface. 

Unlike acoustic sensors, most optical sensors are vicinity sensitive that means that substances do not need to be bound to the surface to be detected but within the penetration depth of the evanescent wave. Both substances bound to the sensor surface and substances that are close to the surface as well of changes in the optical properties (e.g., solvent concentration) of the liquid cause a signal. Bound and unbound substances can be distinguished using a reference channel with a passivated surface. 

#### 2.2.1. Surface Plasmon Resonance (SPR)

The SPR is the transducer with clearly the highest market share in the BIA market. This can be accredited to the high sensitivity of the technique [57], but also to the successful marketing concept of the leading vendor Biacore (GE Healthcare, Uppsala, Sweden) [58] and their high investments into the development of the technology [59] and especially their sensitivity-increasing dextran matrix surfaces [60]. 

Surface plasmons are oscillations of the free electron density in e.g., a metal. These plasmons can be excited when polarized light is diffracted on an interface between a dielectric and some metals at the angle of total reflection, with gold being the most commonly metal used for BIA. The angle of total reflection depends on the refractive index of the surrounding media within the decay length of that electromagnetic wave (called evanescent wave). One way to readout the sensor signal is to measure the intensity of the reflected light for different angles. At the angle where the plasmons are excited, energy is adsorbed and the intensity of the reflected light has a minimum. This angle depends on the amount of mass adsorbed at the surface. 

**Figure 4:**
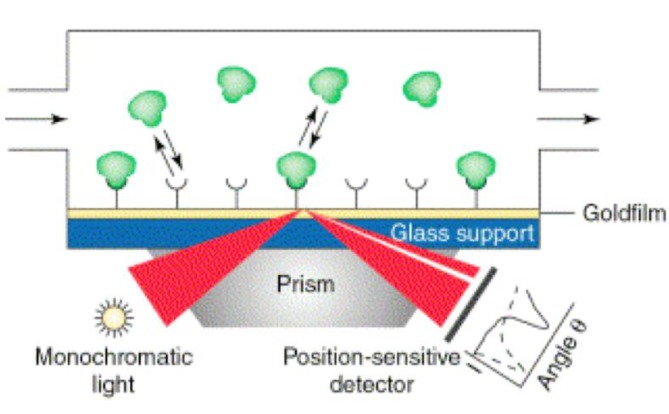
Schematic diagram of the surface plasmon resonance (SPR). Monochromatic light is reflected on a gold surface. At a certain angle, where the surface plasmons are excited, the reflected light has a minimum, which is continuously measured. This angle is directly connected with the analyte bound to the surface. Reprinted from [1] with permission from Elsevier.

Figure 4 shows the set-up of an SPR sensor: The light emitted by a monochromatic light source is reflected at the interface between gold and liquid surrounding. The reflected light is detected and analyzed. As an alternative to the prism shown in the figure, the light can be coupled in using an optical grating. As an alternative for reading out the angle, the wavelength or the intensity at a certain angle can be measured. However, a prism coupler in combination with reading out the angle with a minimum in intensity is the mostly widely used as it has the highest sensitivity [61]. 

A more detailed overview about the SPR technology can be found in [6]. Even though the SPR requires a metal surface, many other functional layers can be put on top, e.g., the carboxymethylated dextran surface introduced in 1990 by Löfås *et al*. [61]. One limitation of the SPR technology might be the substantial cost especially for systems with higher number of sensors usable in parallel like the Biacore 4000 with 20 individually accessible sensors in 4 different flow cells [62]. 

#### 2.2.2. Surface Plasmon Resonance Imaging (SPRi)

**Figure 5:**
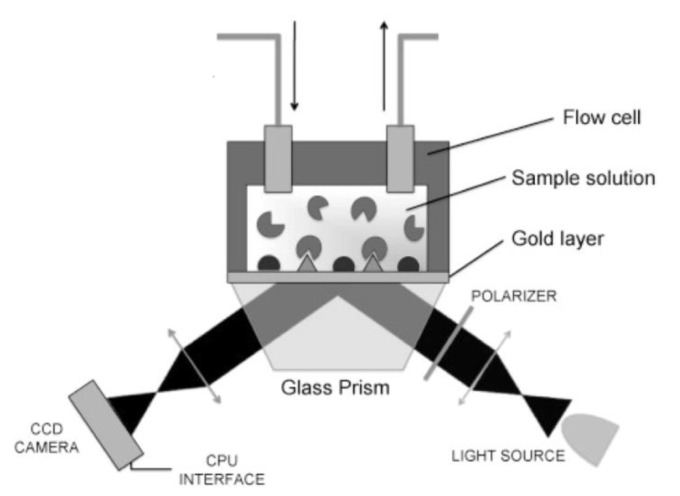
Surface plasmon resonance imaging (SPRi) setup: Instead of measuring at one spot like with the SPR, the reflection of a number of spots is measured using a CCD camera. Reproduced from [68] with permission from Elsevier.

The SPRi technology enables to build microarrays based on SPR. In order to measure multiple sensitive spots using the same set-up, a CCD camera is used to record the intensity of the reflected light at a fixed incident angle and wavelength (Figure 5). Due to the higher complexity of this technique, the SPRi systems have a somewhat lower sensitivity than the SPR [63]. However, the published detection limit of 0.1–1 ng/cm^2^ is sufficient for e.g., DNA [64] and protein [65] detection. 

The number of parallel measurements in the literature is in the range of thousands but the possible number of sensors on an area of 1.4 cm^2^ has been estimated to be more than 10,000 [66]. The number of sensitive spots is basically only limited by the available area and the number of individually accessible spots. While a high number of different substances can be easily immobilized by addressing single spots e.g., using a microspotter [67], it is difficult to access the functionalized spots with different ligand solutions. 

#### 2.2.3. Biolayer Interferometry (BLI)

**Figure 6:**
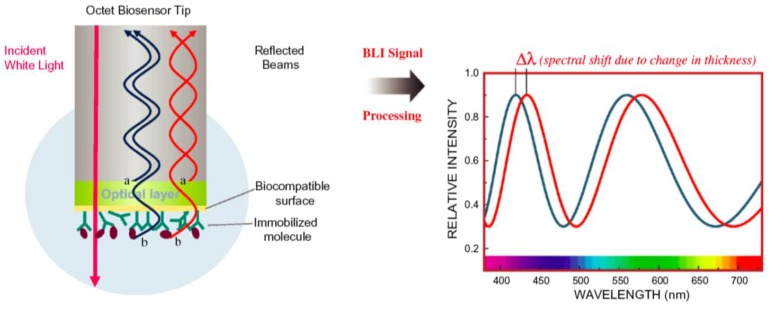
An optical fiber used for Bio-Layer Interferometry and a typical optical signal. Reproduced from [70] with permission from Elsevier.

BLI uses white light interferometry, a rather old technique commonly used to measure the thickness of transparent thin-films [69], to quantify the biomolecules adsorbed to the end of optical fibres. White light travelling through an optical fiber is reflected at the two surfaces: At the fiber-biomolecular layer interface and at the biomolecular layer—buffer interface. The reflected beams interfere generating a signal that directly depends on the amount of adsorbed molecules (Figure 6) [70]. 

The set-up using optical fibers makes an innovative sample delivery system possible: Instead of using a fluidic system to deliver the sample liquids to the sensor, the sensors (*i.e*., the optical fibres) are moved and dipped into well plates. A measurement sequence is performed by dipping the sensors into different reagent solutions. This makes the use of a fluidic system obsolete, which adds robustness to the systems and decreases maintenance and operating costs. Flow can be created, e.g., for diffusion limited reactions or to reduce rebinding when measuring off rates, by shaking the well in an orbital motion. Up to 16 sensors can be used in parallel by the Octet system (ForteBio, Menlo Park, CA). Because only substances bound to the sensor surface are detected, there is little influence from the media surrounding the sensor and thus no reference channel is needed. The downside of the BLI might be the limit of detection of around 0.1 ng/cm^2^, which makes it difficult to follow the adsorption of small molecules [71]. 

#### 2.2.4. Diffraction Grating Based Sensors

**Figure 7:**
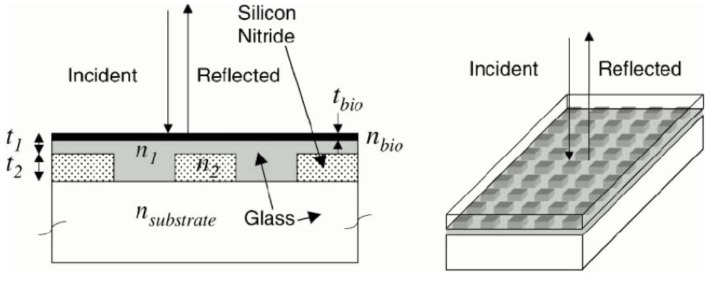
Schematic diagram of the photonic crystal used for colorimetric resonant reflection. Reproduced from [72] with permission from Elsevier.

Diffraction grating based sensors measure the reflection of light on a photonic crystal. A photonic crystal is an optically regular structure made of a dielectric material, e.g., a grating comprising holes and spaces in the nanometer dimension (Figure 7). Such a grating has been presented for usage as a biosensor [72]. When white light is radiated onto the grating, light of only a single wavelength is reflected. The wavelength of this light changes when biomolecules adsorb to the surface of the photonic crystal. For this type of photonic crystal a detection limit for protein of around 0.1 ng/cm^2^ has been reported [13]. 

The advantage of this technology lies in the cheap manufacturing process and the resulting possibility to build highly multiplexed sensor. SRU Biosystems, Inc. (http://www.srubiosystems.com) commercialized this technology under the name BIND™. They provide the sensors in microplates with 96-, 384- and 1,536-well formats. 

#### 2.2.5. Optical-Waveguide-Based Transducers

In optical-waveguide-based biosensors, light is coupled into an optical waveguide. When the incident beam fulfils the condition of total reflection, the light forms a standing wave inside the waveguide, whose eigenvalues depend on the boundary conditions of the waveguide. The intensity of the coupled light depends on the refractive index and the thickness of the layer of biomolecules adsorbed to the surface of the waveguide [73,74]. This waveguide has to be transparent with a refractive index higher than the surrounding media and the thickness has to be around the wavelength of the incident light. Dielectric metal oxides (TiO_2_, Ta_2_O_5_, SiO_2_, ZrO_2_, Nb_2_O_5_) have been used as coatings because of their high refractive index and because they are corrosion resistant in buffer solutions. With the use of a conductive coating such as indium doped tin oxide (ITO) optical-waveguide-based biosensors can be combined with an electrochemical sensor which increases the spectrum of possible applications of this technology [75]. 

**Figure 8:**
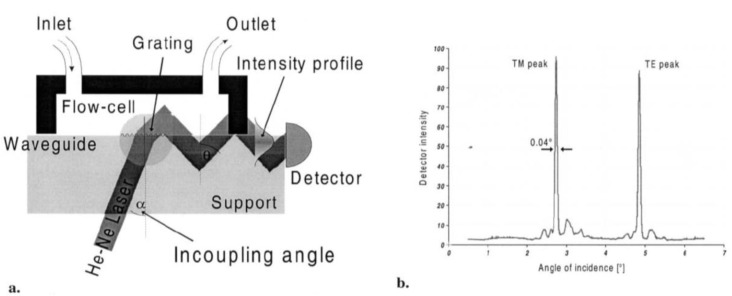
Working principle of Optical waveguide lightmode spectroscopy (OWLS): (**a**) Light is coupled into an optical waveguide via an optical grating and the intensity is measured as a function of the incident angle. From the two peaks in the intensity spectrum (incoupling angles) (**b**), the thickness and the refractive index of the adsorbed layer can be calculated. (Reproduced from [76] with permission from Elsevier).

There are a range of different optical-waveguide-based biosensors that differ in the way the light is coupled into the waveguide and the way the coupled light is detected: The light can be coupled into the waveguide using an optical grating, or by putting the light source directly in line with the wave guide. Also the coupled light can be guided to the detector using a grating or directly. As an example, with Optical Waveguide Lightmode Spectroscopy (OWLS) the light is coupled into the waveguide using a grating and is detected directly (Figure 8). A comprehensive review about theory, methods and applications can be found in [5]. 

If the refractive index of the adsorbed material is known the thickness of the adsorbed material can be calculated. Otherwise, measuring both the transverse electric (TE) and transverse magnetic (TM) modes is required in order to calculate the refractive index and the thickness. While the capability of measuring the refractive index and the film thickness at the same time is an advantage, the sensitivity might be the main disadvantage. The limit of detection of OWLS has been reported to be 0.5 ng/cm^2^ [76]. 

With Dual-Polarization Interferometry (DPI) the light goes through two waveguides, one is for reference without liquid contact, the other is in contact with the liquid surrounding. After exiting from the waveguide, the light is allowed to interfere. As one of the light beams has undergone a phase shift because of the contact with the liquid surrounding, the amount of adsorbed biomolecules can be determined from the interference pattern [77,78]. 

#### 2.2.6. Ellipsometry (ELM)

**Figure 9:**
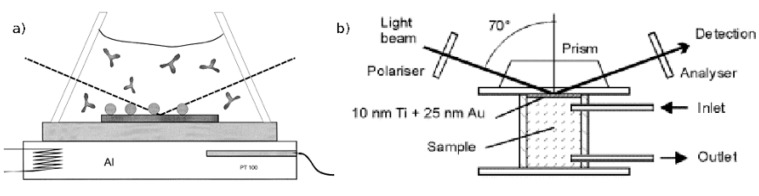
Set-up of the ellipsometry (ELM) (**a**) and the surface plasmon enhanced ELM (**b**). Reproduced from [81,82] with permission from Elsevier.

Ellipsometry (ELM) is a technique that measures the changes in the state of polarization of elliptically polarized light, which is reflected at planar surfaces (Figure 9) [79]. If the available measurement data is very accurate, both the refractive index and the thickness of the adsorbed layer can be obtained from the changes in the ellipsometric angles [80]. Assuming that the refractive index of protein films is around 1.5 the film thickness can be calculated more easily [76]. The complex theory behind the calculations, especially if systems with unknown optical properties are investigated, together with the requirement of reflecting surfaces might be named as main disadvantages of this technique. 

Imaging ellipsometry has been reported to allow measuring more than 105 pixels on an area of less than one cm^2^ in one second. For this technique a CCD camera was used as a detector. ELM allows determining the thickness of solid thin-films in air with accuracy well below 1 Angstrom, the detection limit for the adsorption of biomolecules is average: A detection limit of around 1 ng/cm^2^ has been reported for surface plasmon enhanced ellipsometry [81]. 

### 2.3. Isothermal Titration Calorimetry (ITC)

**Figure 10:**
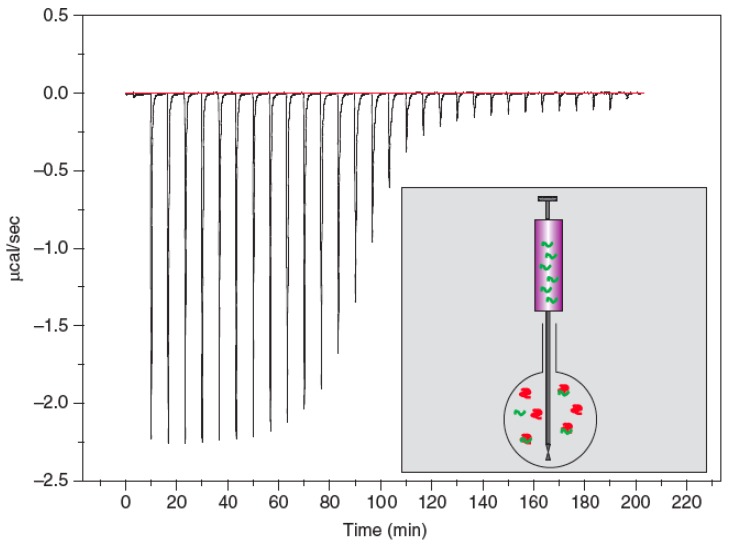
Isothermal titration calorimetry (ITC) setup (inlet) and typical measurement curve: the raw data and the isothermal. Reproduced from [86] with permission from Elsevier.

In isothermal titration calorimetry (ITC) (Figure 10) a solution of one type of biomolecule is titrated into the solution of a binding partner and the heat adsorbed or generated by the biochemical reaction is measured. From the heat of reaction for different concentrations the binding constant, K, the number of binding sites or the stoichiometry (n) and the thermodynamic data, the enthalpy (ΔH_) and entropy (ΔS) of the binding, can be determined in a single measurement. 

Being able to measure heat effects as small as 0.4 μJ (0.1 μcal) allows the determination of binding constants, K’s, as large as 10^8^ to 10^9^ M^–1^. The typical setup consists of a sample and a reference cell in a thermostatted environment, a syringe to introduce the ligand solution into the sample cell, a means to keep the sample cell at the same temperature as the reference cell and to measure the heat changes. The cell volume is typically in the ml range and the injected volume can range from about 1 to 20 μL [83]. 

The high number of parameters that can be measured at the same time and the fact that the reaction can be performed in solution and neither a label nor the immobilization on a surface is needed are the unique features of this technique. The high experimental effort for planning and performing the measurement and the high sample consumption are the drawbacks of this technique. 

A related technique is the differential scanning calorimetry (DSC), where the temperature of a biomolecular solution is changed and the resulting heat change is measured. This gives information about e.g., conformational changes of proteins [84,85]. 

### 2.4. Electrochemical Sensors

Electrochemical sensors are of high importance for the biosensor market. While this section can only give a rough overview, the interested reader is referred to [8] for a more complete review. Label-free electrochemical sensors are based on measuring the change in charge, current, potential or conductivity that takes place when the target molecule binds to the functionalized sensor surface. 

Also the conductivity of the solution can be measured, as many reactions produce or consume electrons and thereby alter the overall electrical conductivity of the solution. However, because the conductivity of a solution depends on all present ions, this sensing principle is considered to be rather non-specific. Amperometric biosensors measure a change in current. However, many biomolecules like proteins are not electroactive, and therefore a label is required [87]. 

**Figure 11:**
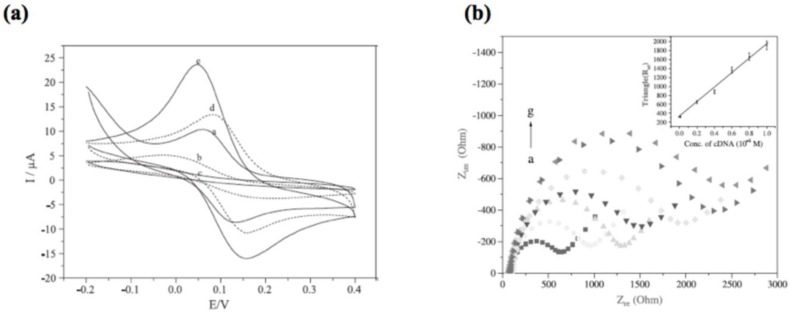
Examples for a measurement curve of cyclic voltammetry (CV) (**a**) and electrochemical impedance spectroscopy (EIS) (**b**). The adsorption of molecules to the surface can be seen from a decrease in current (CV) and an increase in impedance (EIS). Reproduced from [101] with permission from Elsevier.

Impedance sensors measure the electrical impedance between an electrode and the solution at a fixed or variable frequency. The latter approach is called electrochemical impedance spectroscopy (EIS). On adsorption of the target molecule to the electrode, the impedance undergoes a detectable change, which has been shown for a variety of chemical systems [88]. In cyclic voltammetry (CV), the applied voltage is changed at a low velocity and the resulting current is measured. A change in current represents a change of electron transfer resistance using a redox couple such as ferri/ferrocyanide. Molecules adsorbed to the surface act as insulator and increase the resistance Figure 11(a) shows CV curves for a bare gold electrode (a), the adsorption of protein A (b) and IgG (c) at a scan rate of 50 mV/s. Figure 11(b) shows the corresponding EIS measurement. Plotted is the real part *versus* the imaginary part of the electrical impedance from a frequency range from 100 kHz to 0.1 Hz. 

This makes it easy to immobilize a high number of substances (e.g., proteins) and investigate their interaction with one or few ligands (e.g., small molecules) but difficult the other way round. The fact that a wide range of measurements requires the immobilization of few ligand targets and test them against a high number of molecules like in drug discovery motivates developments towards the possibility of accessing a high number of pixels individually in flow [89,90]. 

Electrochemical sensors based on field effect transistors (FET) consist of a transistor where the metal gate is replaced with an appropriate functionalization. On adsorption of the target molecule the potential at the gate oxide changes resulting in a measurable signal between source and drain [91]. One hindrance to commercial success of FET based biosensors apart from the high cross-sensitivity e.g., to changes in pH might be the unsolved challenge to incorporate a high quality but economic reference electrode [87]. 

Electrochemical sensors can be combined with other label-free transducers by integrating a conductive electrode to the setup. This has been shown e.g., for OWLS [92,93], SPR [94,95], ELM [96,97,98], QCM-D [99,100]. These combined set-ups enable to measure the adsorption under applied electric field or to simultaneous measure the adsorption and electrochemically analyze the adsorbent. 

### 2.5. Nanostructure Biosensors

Feynman’s “Plenty of Room at the Bottom” [102] might be valid for label-free biosensors, too. There are nanostructured biosensors emerging that are based on transducers with at least one of the dimensions of the biosensor down to the nanoscale. Along with lower detection limits come a lower liquid and sample consumption and the possibility to integrate a high number of sensors within little space or area. 

#### 2.5.1. Nanoplasmonics

While the conventional SPR uses surface plasmons excited at the interface between a dielectric and a macroscopic gold film, localized surface plasmons resonance (LSPR) can be excited in noble metal nanostructures. These nanostructures can be particles like disks [103], rings [104] or nanoholes in thin-films [105]. They can also be arranged in lines [106] or arrays [106,107]. A peak in the adsorption spectrum appears if the wavelength of the incident phonon is resonant with the localized surface plasmons of conduction electrons of the nanostructure [108]. The position and height of this peak depends on the size, shape and composition of the nanostructure and the local dielectric environment [109,110]. The latter enables to measure the adsorption of molecules on or in between the nanostructures as the adsorption of molecules causes a shift of the peak in the spectrum. 

The small size of the particles allows detecting very low amounts of adsorbents. The combination of detection limits that are comparable to current commercial instruments together with the small size of the particles makes single molecule detection probable [111]. But apart from the sensitivity in terms of signal-to-noise ratio, which is similar to commercially available SPR systems [112], there are other advantages: the sensitivity to bulk refractive index changes is more than one order of magnitude lower, which might make temperature stabilization obsolete and increase the stability towards small changes in organic solvent (e.g., DMSO in the drug discovery process [113]). Additionally, the required set-up is simpler than for the SPR as the light can be irradiated by a white light source at any angle and therefore does not need any prism for coupling [9]. For a successful commercialization the challenge of producing the required nanostructures in a cheap, robust and reproducible way has to be overcome. 

#### 2.5.2. Nanowire Biosensors

Nanowire biosensors are mainly employed as the miniaturization of electrochemical sensors. Biochemically functionalized, they can be used, e.g., for AC voltammetry [111] or function as gate in FETs [114] by connecting them between source and drain. Like with electrochemical sensors using thin-films, adsorbed biomolecules change the dielectric environment around the nanowire. Due to the small size of the nanowires and the resulting small surface-to-volume ratio, biomolecules binding to the nanowire results in a significant change in the electrical properties of the nanowire [14]. An increasing sensitivity for smaller nanowire diameter has been shown in theory and experimentally [115]. With the diameters being comparable to the size of the biochemical analytes under analysis [8], extremely high sensitivities up to the detection of a single virus has been shown [116]. Also the multiplexed detection of proteins was demonstrated on a multiplexed nanowire sensor [117]. However, the sensitivity is significantly reduced in solutions with high ion concentrations when the analyte adsorbs at a distance from the nanowire that is higher than the Debye length, because this means that the charge of the analyte is shielded by the ions in solution [118]. Three possible materials for nanowires are carbon nanotubes (CNTs), silicon nanowires (SiNWs) and conducting polymer nanowires (CP NWs). CNTs are interesting because they are mechanically stable and exist structure-dependent both as semiconductor and conductor, so that they might be used for several parts of the FET and the connectors. SiNWs and CNTs have a high tensile strength and Young’s Modulus, however, they are always semiconducting. Both the CNTs and the SiNWs are produced under harsh conditions, so the biochemical functionalization has to be done after the production. This is different for the CP NWs, which can be synthesized at ambient conditions using well-known chemical processes and therefore can be functionalized before or during synthesis. A variety of techniques have been employed to assemble the nanowires into functioning devices: Alignment in electric and magnetic field, lithography, Langmuir–Blodgett techniques or biomolecule mediated self-assembly [119]. 

## 3. Conclusions

A broad variety of transducer principles for BIA have been introduced. These were optical (SPR, SPRi, BLI, Diffraction grating based sensors, waveguide-based sensors, ELM), acoustic (QCM, SAW and FBAR), electrochemical and calorimetric sensors. Their different working principles result in different properties like sensitivity, sample consumption or the ability for multiplexed sensing. While the sensitivities were stated for all transducers it should be kept in mind that it is especially the perceived sensitivity of an operator in daily routine that will count for successful commercialization. For this, in addition to sensitivity, also usability, easy handling, reproducibility and robustness play an important role. 

It is also important to take the state of development of the technology into account. The sensitivity of a sensor technology can change largely in course of time and with higher recourses for research and development. Biacore, for example, improved the surface mass sensitivity by a factor of 20 to 30 and the association constants by nearly three orders of magnitude in only one decade [120]. It can be thus expected that sensors in early development stages can undergo a similar improvement. 

In addition, also other factors such as the possibility to integrate a high number of sensors in one device can play an important role. While the sensor principles with implemented nanostructures have the most promising properties among the presented transducers in terms of sensitivity, sample consumption and number of parallel measurements they might have the longest road to commercialization in front of them. Apart from the potential toxicity of the nanoparticles [121] which have to be taken into account, the problem of producing them in an economic way at high quantities and quality offers lots of interesting working tasks for research and development. 
